# Transcriptional analysis of duck liver infected with duck hepatitis A virus type 1 isolate HA5

**DOI:** 10.3389/fvets.2026.1739363

**Published:** 2026-03-02

**Authors:** Dan Yin, Yuehua Gao, Xiaozhen Guo, Huaiying Xu, Yufeng Li, Feng Hu, Kexiang Yu, Bing Huang, Zhuoming Qin, Jingchao Yang, Xiuli Ma

**Affiliations:** 1 Shandong Provincial Key Laboratory of Livestock and Poultry Breeding, Institute of Poultry Science, Shandong Academy of Agricultural Sciences, Jinan, China; 2 Shandong Animal Husbandry General Station, Jinan, China

**Keywords:** DHAV-1, differential expression, ducklings, liver injury, mRNA-seq

## Abstract

Duck Hepatitis A Virus Type 1 (DHAV-1) is a major pathogen in ducklings, characterized by severe hepatomegaly and punctate hepatic hemorrhage. In this study, we investigated host gene expression dynamics in specific-pathogen-free (SPF) ducklings infected with the DHAV-1 isolate HA5 using high-throughput RNA sequencing (RNA-seq). We performed comprehensive transcriptomic analyses, integrating Clusters of Orthologous Groups (COG) classification, Gene Ontology (GO) annotation, and Kyoto Encyclopedia of Genes and Genomes (KEGG) pathway enrichment. By combining these data with viral replication kinetics, we aimed to elucidate the molecular mechanisms of DHAV-1 pathogenesis. Differentially expressed genes (DEGs) were identified at 6, 12, 24, and 48 h post-infection (hpi). Viral titers peaked at 24 hpi and declined by 48 hpi, correlating with the observed transcriptional changes. The most pronounced transcriptional response occurred at 24 hpi, with 4,067 DEGs detected. Functional enrichment analyses revealed that these DEGs were predominantly associated with immune and metabolic pathways, including the Jak–STAT signaling pathway, oxidative phosphorylation, and Toll-like receptor signaling pathway. Collectively, these findings highlight the complex interplay between host immune responses and metabolic reprogramming. This study provides novel insights into the molecular basis of DHAV-1-induced liver pathology.

## Introduction

Duck viral hepatitis (DVH) primarily arises from infection by the duck hepatitis A virus (DHAV), a highly virulent pathogen that presents a substantial risk to the global duck farming industry. Predominantly affecting ducklings less than three weeks old, this disease exhibits high contagion and rapid transmission, characterized clinically by opisthotonos and hemorrhagic liver lesions (1, 2). In the absence of effective control measures, DVH can result in up to 90% mortality among infected ducklings (3). Three genotypes of DHAV (DHAV-1, DHAV-2, and DHAV-3) have been distinguished through serological and phylogenetic analysis (4). DHAV-1 is prevalent in several regions globally, including Poland, England, Egypt, and China, following its emergence in the United States (5). In contrast, DHAV-2 remains geographically confined to Taiwan, China (6). DHAV-3, a novel genotype identified in Korea in 2003, subsequently spread to China, Vietnam, and recently to Egypt (7). On a global scale, DHAV-1 is the sole genotype present in North America and Europe, with occurrences in the United States, England, and Poland. DHAV-1 is widely distributed across the world and represents a significant barrier to the advancement of the duck industry, particularly in eastern Asia (8, 9).

DHAV virions exhibit a spherical morphology devoid of an envelope, featuring an icosahedral capsid organized into 12 pentameric units (7). DHAV-1, a prototypical picornavirus, is characterized by its single-stranded positive-sense RNA genome. Spanning approximately 7,700 nucleotides, this genome comprises a 5′ untranslated region, an open reading frame, and a 3′ untranslated region, culminating in a poly(A) tail (10, 11). The pervasive threat posed by DHAV-1 to duckling populations underscores the imperative to elucidate its molecular pathogenesis.

The interactions between virus and host are profoundly influenced by the pathogen’s virulence and the host’s immune response, which can precipitate alterations in host gene expression (12). DHAV-1 is known to induce characteristic liver lesions in ducklings. Despite advancements, the intricacies of DHAV-1-host interactions remain largely elusive, with scant information available on the gene expression changes in duckling liver cells in response to DHAV-1 infection. The transcriptome encompasses all gene transcription products of a specific tissue or cell in a functional state, serving as a critical link between genomic information and proteomic functions. Transcriptomic analyses form the foundation for studying gene function and regulatory networks. Increasingly, transcriptome sequencing is recognized as a potent tool for elucidating the molecular underpinnings of host-virus interactions (13, 14). To date, numerous studies have reported on transcriptome sequencing following DHAV-1 infection in ducks. For instance, one study delineated mRNA and miRNA profiles in DHAV-1-infected duck embryo fibroblast (DEF) cells via RNA sequencing (RNA-seq) and miRNA sequencing (miRNA-seq) (15). Another investigation compared mRNA and miRNA expression patterns in DHAV-1-infected duckling livers at 16 h post-infection (hpi) to elucidate the underlying mechanisms and dynamic changes (16). Nonetheless, transcriptome sequencing at additional time points in DHAV-1-infected duckling liver cells remains unexplored.

In this study, we conducted a comparative analysis of transcriptome data from the livers of ducklings infected with lethal DHAV-1 at various time points post-infection. Sequenced segments were analyzed to identify differentially expressed immune-related genes using the Clusters of Orthologous Groups (COG), Gene Ontology (GO), and Kyoto Encyclopedia of Genes and Genomes (KEGG) databases. Selected differentially expressed genes (DEGs) were validated through reverse transcription quantitative polymerase chain reaction (RT-qPCR). This research lays a foundation for understanding the pathogenesis of DHAV-1 infection and may aid in identifying candidate genes that confer resistance to DHAV-1 in ducks. Additionally, it enhances our comprehension of the intricate virus-host interactions.

## Materials and methods

### Virus and animals

The DHAV-1 HA5 strain utilized in this study was isolated from a one-week-old duckling in China, which exhibited characteristic clinical symptoms and pathological changes of DVH. The HA5 strain was propagated in eleven-day-old embryonating specific-pathogen-free (SPF) duck eggs and subsequently stored at −80 °C for further experiments. The SPF duck eggs were sourced from Shandong Haotai Experimental Animal Breeding Co., Ltd. Additionally, one-day-old SPF shelducks were procured from the same supplier and maintained in isolators until use.

### Viral infection and sample collection

Thirty two-day-old SPF shelducks were randomly selected and intramuscularly inoculated with a lethal dose of the DHAV-1 HA5 strain, each receiving 3 × 10^5^ ELD_50_. At 6, 12, 24 and 48 hpi, three ducklings from the infected group were euthanized, and fresh liver tissue samples were collected. A control group, treated with an equivalent dose of isotonic sodium chloride. For each time point, there were three independent biological replicates, with one duckling per replicate, and no pooling of samples was performed. All liver tissues were immediately washed with ice-cold PBS, frozen in liquid nitrogen, and stored at −80 °C until processed for total RNA extraction for RT-qPCR and transcriptome sequencing analysis.

### Determination of replication kinetics of the DHAV-1 HA5 strain

Liver tissue samples collected at various time points were subjected to ten-fold serial dilutions in sterile phosphate-buffered saline containing antibiotics. Following incubation, allantoic fluid was collected from both dead embryos and those surviving for 5 days post-inoculation. Embryos were examined for the presence of characteristic lesions. The 50% embryo infectious dose (EID₅₀) was determined according to the method of Reed and Muench.

### mRNA sequencing

mRNA sequencing was performed by Biomarker Technologies Co., Ltd. (Beijing, China). Total RNA from three ducks per group was extracted using TRIZOL reagent (Takara, China) according to the manufacturer’s protocol. The quantity and quality of the RNA were assessed, and only high-quality RNA was selected for library construction. Strand-specific RNA-Seq libraries were prepared using the Illumina Stranded mRNA Prep kit, ensuring the preservation of transcriptional directionality. Each library contained an average 300 bp cDNA insert flanked by adapter sequences. Sequencing was then carried out on an Illumina HiSeq 2,500 platform (Illumina, Inc.; San Diego, CA, USA) in 125 bp paired-end (PE125) mode. A typical coverage of at least 20–30 million clean reads per sample was achieved to ensure robust quantification of gene expression.

### Analyses of RNA-seq data

To ensure the reliability of the analyzed data, raw sequencing reads underwent rigorous filtering to eliminate low-quality and contaminated sequences, yielding a set of clean reads. Quality assessment of the filtered data was conducted using FastQC (v0.11.9) (17). The high-quality sequences were aligned to the *Anas platyrhynchos* reference genome (GenBank assembly accession: GCF_015476345.1, ZJU1.0) using the STAR (v2.7.9a) (18). We utilized the NCBI *Anas platyrhynchos* Annotation Release 104. However, we noted that potential gaps in microchromosome assembly and GC-rich promoter regions remains a known limitation in the duck genome which may impact the detection of low-abundance transcripts. Transcript reconstruction was carried out with Cufflinks software (19). The expression of each gene was calculated according to the reads per kilobase per million reads (RPKM). DEGs were screened using DESeq2 (v1.26.0), with selection criteria set at a false discovery rate (FDR) < 0.01 and |log2FoldChange| > 1 (20). The log2FoldChange threshold was applied globally across all time points, and DEGs were then compared and tracked across different time points for downstream analyses to observe temporal expression patterns. Functional enrichment analyses (COG, GO, and KEGG) were executed using clusterProfiler. In order to control the errors in these large-scale comparisons, multiple-testing correction was applied using the Benjamini-Hochberg (BH) procedure. GO terms and KEGG pathways were considered significantly enriched only if the adjusted *p*-value was < 0.05.

### RT-qPCR validation

The sequencing results of selected mRNAs were validated using RT-qPCR. Primers for RT-qPCR were designed based on sequences obtained from the NCBI database (Table 1). Total RNA was extracted from the collected tissue samples using MolPure® TRIeasy Plus Total RNA Kit (Cat No. 19211; Yeasen, Shanghai, China) and quantified with a Nanodrop spectrophotometer (Thermo Fisher Scientific, USA). cDNA synthesis was performed using the HiScript® II RT Supermix for qPCR Kit (Vazyme, China) following the manufacturer’s protocol. RT-qPCR reactions were carried out in a 20 μL volume using the LightCycler (Roche Diagnostics, Mannheim, Germany) and the SYBR Green PCR Kit (Takara, Dalian, China) according to the manufacturer’s instructions. The PCR cycling conditions were set as follows: an initial denaturation at 95 °C for 30 s, followed by 40 cycles of denaturation at 95 °C for 5 s, and extension at 60 °C for 30 s. Each sample was analyzed in triplicate.

**Table 1:** Primers used in this study.

Name	Sequence of primers (5′-3′)
MDA5	F: CTGCCCGCTACTTGAACTCCA
	R: GCACCATCTCTGTTCCCACGA
IL-12	F: TACACCTGCCTGTCTGCT
	R: CGTCTTGCTTGGCTCTTT
IFN-α	F: TCCACCTCCTCCAACACCTC
	R: TGGGAAGCAGCGCTCGAG
TLR7	F: CCTTTCCCAGAGAGCATTCA
	R: TCAAGAAATATCAAGATAATCACATCA
IL-8	F: AAAAGCACAATGGGTCTC
	R: TGCCGTTCATGGTTAGGA
IL-10	F: AGCAGCGAGCACCACCA
	R: TGCCGTTCTCGTTCATCTTT
GAPDH	F: ACATCATCCCTGCCTCTACTG
	R: CCTGCTTCACCACCTTCTTG

For statistical analysis, the relative expression levels of target genes in the infected and control groups were calculated using the 2^−∆∆Ct^ method. Expression levels were normalized to the housekeeping gene GAPDH, which served as an endogenous control, and results were expressed as fold changes in gene expression.

## Result

### Replication of DHAV-1 HA5 strain in liver tissue

**Figure 1:**
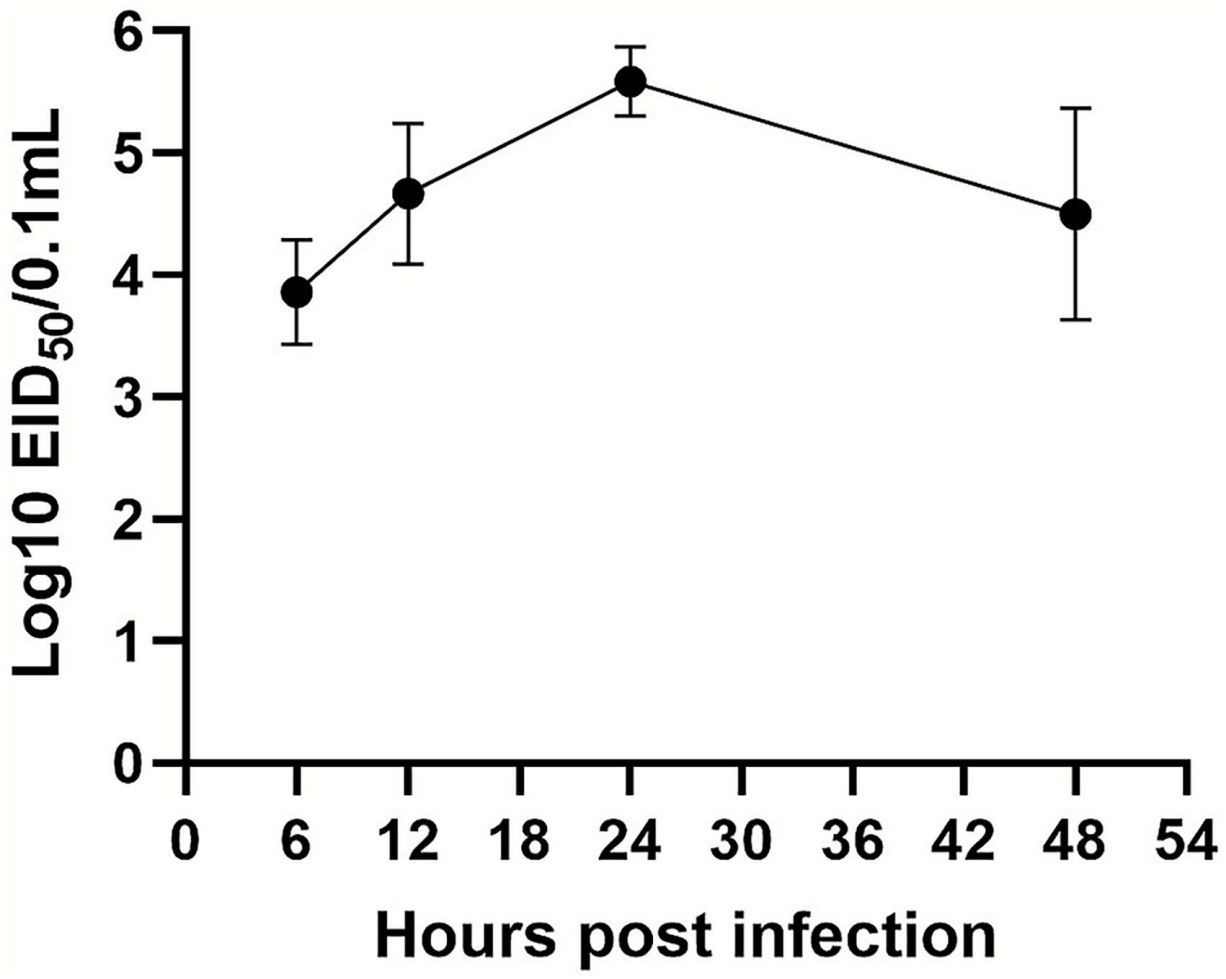
Growth kinetics of the DHAV-1 HA5 strain in liver tissue. Fresh liver tissue samples were collected from SPF ducks infected with the DHAV-1 HA5 strain at 6, 12, 24, and 48 hpi for EID_50_ determination. Data are presented as means ± SD from three independent experiments.

EID_50_ of DHAV-1 in duck liver was measured at various time points following infection with the DHAV-1 HA5 strain. The viral titer increased progressively from 6 hpi, peaking at 24 hpi, before beginning to decline (Figure 1).

### Transcriptional profiling and DEGs analysis

We conducted transcriptional profiling of livers infected with the HA5 strain. RNA-seq was employed to analyze the total RNA from the livers of SPF shelducks infected with the HA5 strain at various time points, as well as from mock-infected controls. The DEGs identified (FDR < 0.01 and |log2FoldChange| > 1) are listed in Supplementary Table S1.

**Figure 2:**
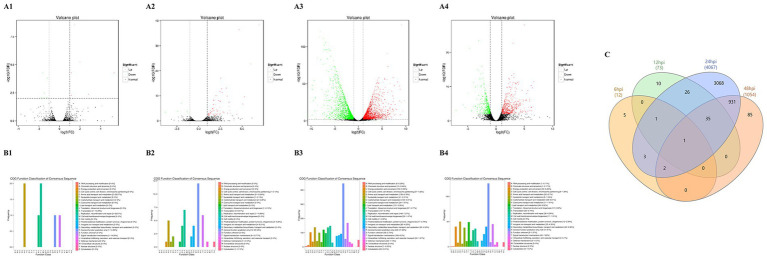
Volcano, venn, and COG function classification of DEGs between infected groups and control groups. **(A1–A4, B1–B4)** Correspond to volcano plots and COG function classification, respectively. Specifically, panel **(A1,B1)** illustrate the comparison between the groups at 6 hpi, panel **(A2,B2)** at 12 hpi, panel **(A3,B3)** at 24 hpi, and panel **(A4,B4)** at 48 hpi. In the volcano plots, red points indicate upregulated genes, while green points represent downregulated genes. These analyses were conducted by comparing DEGs in infected ducklings with those in control ducklings. **(C)** Venn diagram of differentially expressed genes.

At 6 hpi, 12 DEGs were identified, with an equal distribution of 6 upregulated and 6 downregulated genes. By 12 hpi, the number of DEGs increased to 73, comprising 67 upregulated and 6 downregulated genes. The most substantial transcriptional response was observed at 24 hpi, with 4,067 DEGs, including 1981 upregulated and 2086 downregulated genes. At 48 hpi, the number of DEGs decreased to 1,054, with 543 upregulated and 511 downregulated genes (Table 2). The progressive increase in DEGs during the course of DHAV-1 infection, peaking at 24 hpi, suggests that the infection intensifies between 12 to 24 hpi and then begins to subside after 24 hpi. The upregulated and downregulated genes are depicted in the volcano plots (Figures 2A1–A4). To distinguish between transient and sustained host responses, a cross-time-point overlap analysis was conducted (Figure 2C). We identified 35 genes that were consistently differentially expressed across the 12, 24, and 48 hpi time points. This persistent core signature was predominantly composed of genes involved in innate immune signaling and antiviral defense (e.g., IRF3, MX, CCL4 and IFIT5), indicating a sustained attempt by the host to suppress DHAV-1 replication.

**Table 2:** Overview of differential gene expression analysis results.

Comparison Group	Total DEGs	Up-regulated	Down-regulated
6 hpi vs. control	12	6	6
12 hpi vs. control	73	67	7
24 hpi vs. control	4,067	1981	2086
48 hpi vs. control	1,054	543	511

### COG classification of DEGs

DEGs were annotated using the COG database to determine the biological processes involved at each time point. At 6 hpi, the COG analysis revealed that most DEGs were involved in amino acid transport and metabolism, translation, ribosomal structure, and biogenesis (Figure 2B1). These findings suggest early alterations in protein synthesis and metabolic adaptation, likely as an immediate host response to viral entry.

By 12 hpi, the range of affected functional categories expanded significantly. Key processes included amino acid transport and metabolism, transcription, replication, recombination and repair, inorganic ion transport and metabolism, general function prediction only, and signal transduction mechanisms (Figure 2B2). The involvement of transcriptional and replication-related processes indicates the host’s attempt to modulate gene expression and potentially manage viral replication.

At 24 hpi, the host response became more complex. DEGs were associated with energy production and conversion, amino acid transport and metabolism, lipid transport and metabolism, translation, ribosomal structure, and biogenesis, transcription, replication, recombination and repair, secondary metabolites biosynthesis, transport and catabolism, general function prediction only, and signal transduction mechanisms (Figure 2B3). This broad range of functional categories suggests a multifaceted host response, involving metabolic shifts, cellular stress responses, and signal transduction pathways.

The gene expression profile at 48 hpi (Figure 2B4) closely mirrored that of 24 hpi, indicating that the host had settled into a sustained response pattern. The persistence of DEGs in categories such as energy production, amino acid and lipid metabolism, and signal transduction mechanisms suggests ongoing efforts to maintain cellular homeostasis and manage viral load.

### GO analysis

To elucidate the functional roles of the DEGs, GO analysis was conducted. This analysis categorized and annotated the DEGs into three primary domains: biological processes, cellular components, and molecular functions.

**Figure 3:**
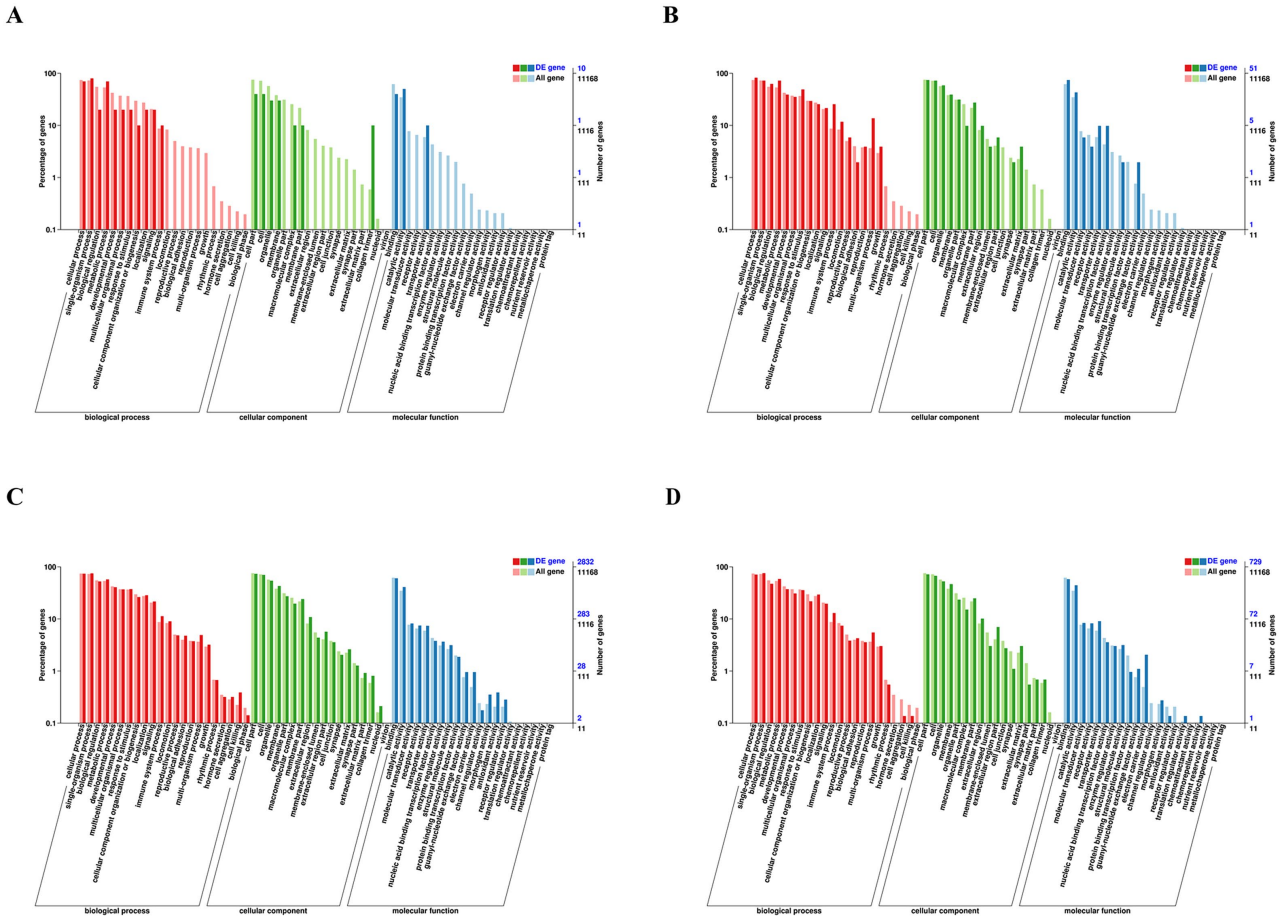
GO classification analyses of DEGs expressed at 6 hpi **(A)**, 12 hpi **(B)**, 24 hpi **(C)**, and 48 hpi **(D)**. GO classification: the abscissa is the GO category, the left ordinate is the percentage of the number of genes, and the right ordinate is the number of genes. This figure shows the gene enrichment of each secondary function of GO terms in the background of differentially expressed genes.

At 6 hpi, the overall number of DEGs was relatively small (Figure 3A). Nevertheless, these DEGs were enriched in biological processes such as cellular process, single-organism process, and metabolic process. For cellular components, the DEGs were mainly associated with cellular parts, cells, organelles, and membranes. In terms of molecular functions, the DEGs were predominantly involved in binding, catalytic activity, and transporter activity.

By 12 hpi, there was an increase in the number of DEGs (Figure 3B). In addition to the categories identified at 6 hpi, there was enrichment in biological regulation, response to stimulus, immune system process, membrane, organelle part, receptor activity, and nucleic acid binding transcription factor activity. This increase indicates a more complex host response, including regulatory and immune-related processes.

At 24 hpi, DEGs were enriched across a broad range of categories in all three GO domains (Figure 3C). This expansion reflects the host’s intensified response to the viral infection, activating various biological processes, cellular components, and molecular functions.

The number of DEGs decreased and stabilized at 48 hpi (Figure 3D). Despite the reduced number, the categories of enriched GO terms remained consistent, indicating a sustained but regulated response. The stabilization suggests the host’s adaptation to the infection, maintaining essential cellular and molecular functions while possibly minimizing damage.

### KEGG pathway analysis

**Figure 4:**
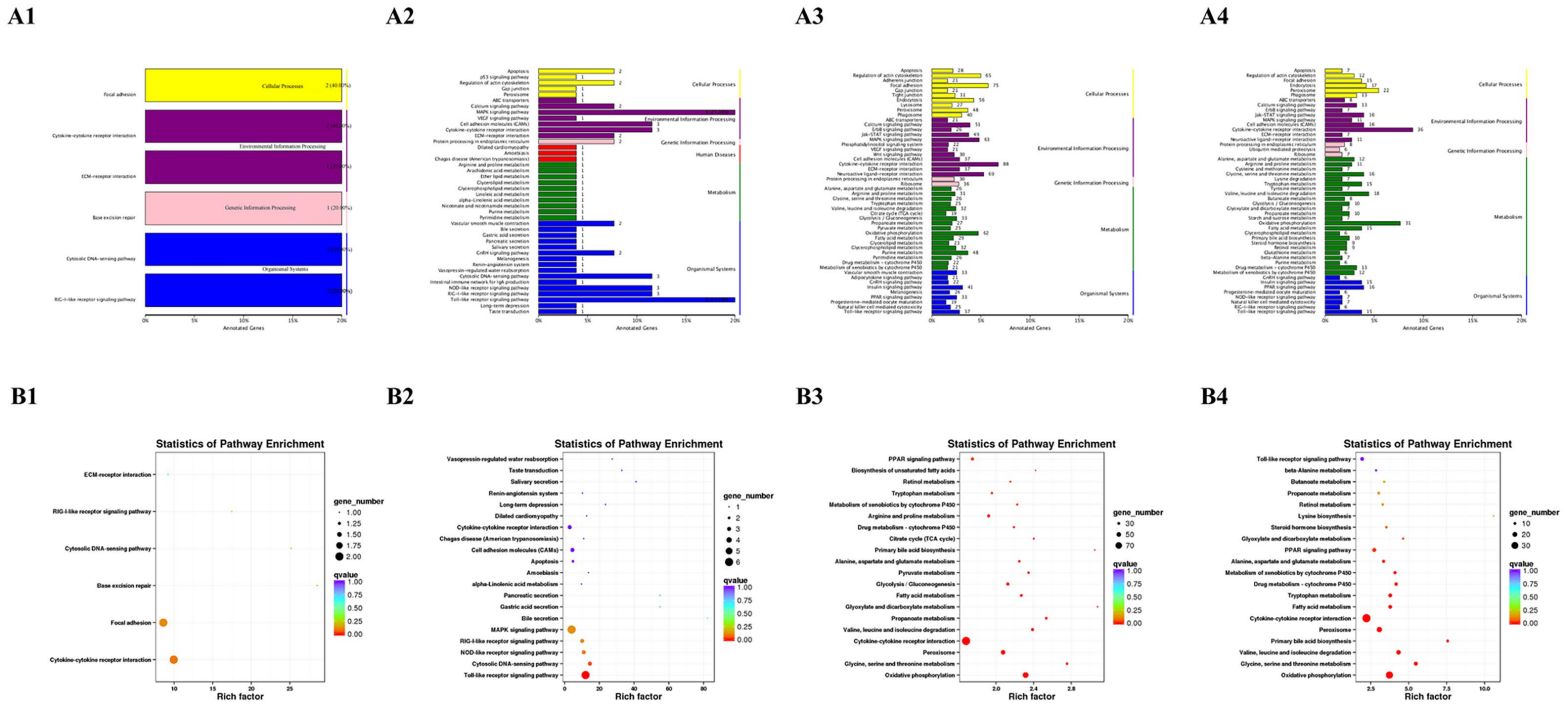
KEGG pathway analysis of DEGs according to 6 hpi **(A1,B1)**, 12 hpi **(A2,B2)**, 24 hpi **(A3,B3)**, and 48 hpi **(A4,B4)**. **(A1–A4)** The ordinate is the name of the KEGG metabolic pathway, and the abscissa is the number of genes annotated to the pathway and the ratio of their number to the total number of annotated genes. **(B1–B4)** Take the KEGG pathway as the ordinate and the rich factor as the abscissa. The size of the dot indicates the number of differential genes annotated to the pathway, and the color indicates the *p*-value significance of the pathway. The figure shows the top 20 KEGG relative enrichment pathways based on *p* value.

At 6 hpi, the DEGs were enriched in pathways related to RIG-I-like receptor signaling, ECM-receptor interaction, cytokine-cytokine receptor interaction, and cytosolic DNA-sensing (Figures 4A1,B1). These pathways are critical for initiating antiviral responses and modulating the immune response. Additionally, significant enrichment in the focal adhesion pathway suggests that early cellular changes occur in both structure and intercellular communication.

By 12 hpi, the immune profile expanded significantly. In addition to the pathways identified at 6 hpi, DEGs showed further enrichment in several critical signaling cascades: MAPK signaling, Toll-like receptor signaling, NOD-like receptor signaling, and cytosolic DNA-sensing pathways (Figures 4A2, B2). The continued involvement of these pathways, underscores a robust and systemic activation of the innate immune response and inflammatory signaling.

At 24 and 48 hpi, the complexity and diversity of DEG involvement increased (Figures 4A3–A4). DEGs were further enriched in pathways associated with Jak–STAT signaling pathway, oxidative phosphorylation, peroxisome, valine, leucine, and isoleucine degradation, fatty acid metabolism, glycine, serine, and threonine metabolism and PPAR signaling pathway. Among these, oxidative phosphorylation and lipid metabolism are particularly relevant to liver pathology. Oxidative phosphorylation plays a critical role in energy production and mitochondrial function, which is often disrupted during viral infections, contributing to liver damage. Lipid metabolism pathways are also crucial, as disturbances in lipid homeostasis can lead to liver steatosis and inflammation, common features of viral-induced liver injury. Furthermore, the Jak–STAT signaling pathway is pivotal in the regulation of immune responses and inflammation. Aberrant activation or dysregulation of this pathway has been linked to chronic inflammation and fibrosis in liver disease. These findings suggest that the dysregulation of these pathways may significantly contribute to DHAV-1-induced liver damage.

### Verification of DEG by RT-qPCR

**Figure 5:**
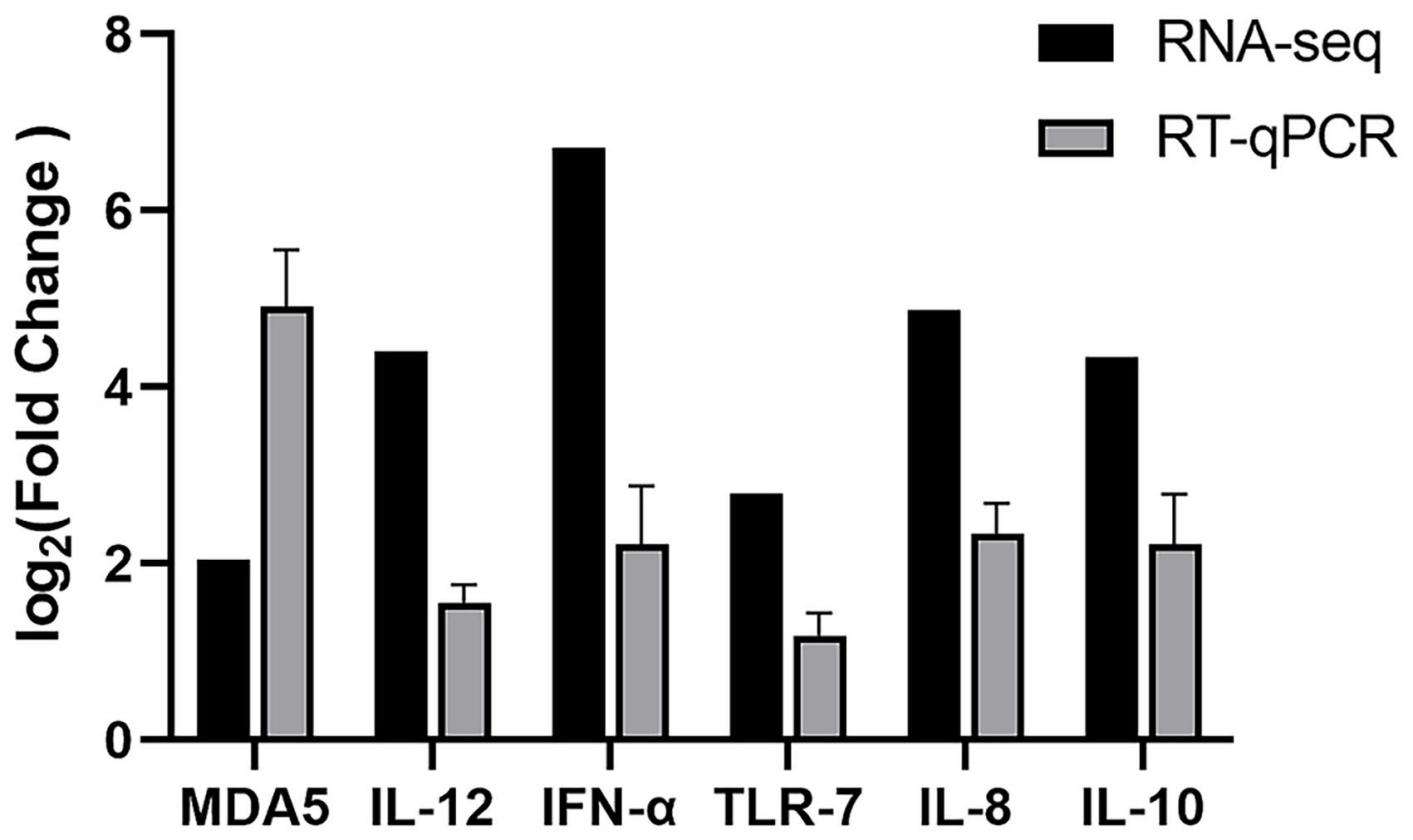
Validation of DEGs by RT-qPCR. The relative expression level of MDA5, IL-12, IFN-*α*, TLR-7, IL-8, and IL-10 in DHAV-1 infected sample was calculated using the 2^−ΔΔCt^ method and represented as the *n*-fold change compared to the mock-infected sample.

We randomly selected six genes (MDA5, IL-12, IFN-*α*, TLR-7, IL-8, and IL-10), which are implicated in RIG-I-like receptor and Toll-like receptor signaling pathways, for validation via RT-qPCR within 24 h post-stimulation. This approach aimed to assess the consistency and reproducibility of the DEGs identified through transcriptome sequencing. The RT-qPCR results confirmed significant changes in the expression of these genes, with upregulation patterns aligning closely with those observed in RNA-Seq data (Figure 5). These findings support the reliability of transcriptome sequencing in identifying DEGs.

## Discussion

DHAV-1 is a virulent pathogen responsible for severe hepatic injury and high mortality rates in young ducks, leading to significant economic losses in the global duck industry (10, 21). Despite its profound impact, the molecular mechanisms underlying DHAV-1 infection remain incompletely understood, and no effective antiviral strategies have been developed to date. Deciphering the molecular interactions between DHAV-1 and its host is therefore imperative. Advances in high-throughput next-generation sequencing (NGS) technologies have emerged as powerful tools for whole-genome viral sequencing, offering unprecedented insights into viral-host transcriptome interactions (14). Several studies have provided foundational transcriptomic data from various tissues of ducks infected with DHAV, reovirus, and duck hepatitis B virus (DHBV), shedding light on the molecular dynamics of viral infections in these hosts (22–24). DHAV-1, known to cause both acute and chronic hepatitis, necessitates a detailed investigation of its effects on host immune responses (25). Prior transcriptomic investigations of DHAV-1 have largely focused on isolated intervals, such as 16 hpi in the liver *in vitro* dynamics in duck embryo fibroblasts (15, 16). Consequently, the longitudinal *in vivo* host response remains poorly characterized. This study provides a comprehensive analysis of the transcriptional landscape in SPF duckling livers across a temporal gradient of 6, 12, 24, and 48 hpi. By integrating host gene expression with viral replication kinetics and progressive histopathology, we delineate the phased transition of the immune response, offering critical insights into the mechanisms governing DHAV-1 pathogenesis.

The integration of COG classification, GO analysis, and KEGG pathway enrichment with viral replication dynamics provides a comprehensive view of the host response to DHAV-1 infection. DHAV-1 infection triggers a complex host response involving immune activation, inflammation, and metabolic changes. The early response (6–12 hpi) is characterized by the activation of innate immune pathways, such as RIG-I-like receptor signaling and cytokine-cytokine receptor interaction, reflecting the host’s attempt to recognize and combat the virus. Previous studies have consistently demonstrated the involvement of RIG-I and MDA5 in the recognition of DHAV-1 infection (26). These pattern recognition receptors are ubiquitously expressed in normal duck tissues and are rapidly and markedly upregulated in response to viral infection (27, 28). In avian models, particularly Fayoumi and Leghorn chickens infected with highly pathogenic avian influenza virus (HPAIV), the “cytokine-cytokine receptor interaction” pathway has been significantly upregulated, underscoring its critical role in modulating the host immune response against HPAIV (29). However, dysregulation of cytokine production can lead to severe tissue damage and, ultimately, host mortality (30). The subsequent activation of MAPK, Toll-like, and NOD-like receptor signaling pathways indicates an escalating inflammatory response, which could contribute to liver damage. These findings highlight the importance of tightly regulated immune responses in combating viral infections while minimizing collateral damage to host tissues.

The peak viral titer at 24 hpi marks a critical point in DHAV-1 infection, where the virus exerts maximum pressure on the host. The concurrent repression of key metabolic pathways suggests that the virus may actively inhibit these processes to redirect cellular resources toward its replication. However, it is also possible that these metabolic alterations reflect secondary hepatocellular damage rather than being purely a viral strategy. The downregulation of oxidative phosphorylation, for example, could indicate a potential energy crisis in the liver cells, impairing their ability to function properly and contributing to liver enlargement. This energy imbalance might be a consequence of cellular stress rather than a direct viral mechanism. Similarly, the suppression of amino acid and fatty acid metabolism may signal broader metabolic dysfunctions, which could be exacerbated by the cellular damage induced by the viral infection. Therefore, while these findings suggest that DHAV-1 may impact metabolic processes to support viral replication, it is important to consider the possibility that these changes are partly a result of hepatocellular injury caused by the virus. This result is similar to the findings of DHAV-3, where the metabolism related processes were strongly inhibited by DHAV-3 infection at 24 hpi, suggesting that DHAV infection can rewire the immune function and metabolism of ducks (1).

The Jak–STAT signaling pathway serves as a pivotal communication hub within the immune system (31). Its activation at 24 hpi underscores the host’s endeavor to mount an effective immune defense. While crucial for restraining viral replication, this response may also incite inflammatory processes that contribute to liver hemorrhage. The inflammatory response plays a crucial and complex role in liver metabolism. While a mild inflammatory response is known to exert consistent hepatoprotective effects, excessive inflammation can lead to liver damage (32). Previous studies have demonstrated that DHAV-1 infection triggers excessive inflammatory responses, resulting in tissue damage in both the liver and kidney (33, 34). These findings suggest that the interplay between viral suppression and immune-mediated injury may be a determining factor in the severity of DHAV-1-induced liver pathology.

Liver enlargement in DHAV-1-infected ducklings may result from a combination of metabolic disruptions and immune-mediated tissue remodeling. The suppression of energy production and lipid metabolism could lead to hepatocyte swelling and steatosis, contributing to hepatomegaly. Simultaneously, the activation of inflammatory pathways through Jak–STAT signaling may promote vascular permeability and hemorrhage, as immune cells infiltrate the liver and release pro-inflammatory cytokines.

By 48 hpi, the decrease in viral titer correlates with a reduction in the number of DEGs, indicating a partial resolution of the infection. However, the sustained activation of immune pathways suggests ongoing liver stress and inflammation, potentially contributing to the observed punctate hemorrhage. The above results suggest that the host response is not only immediate but also sustained, reflecting the ongoing challenge posed by the viral infection.

The observed changes in gene expression and pathway enrichment provide insights into the molecular mechanisms driving DHAV-1-induced liver pathology. The early immune response likely limits viral replication initially, but the subsequent metabolic and inflammatory responses contribute to liver damage and enlargement. Understanding these interactions is crucial for developing targeted therapies to mitigate liver damage and improve the clinical outcomes of DHAV-1 infection.

## Conclusion

The mRNA-seq analysis of DHAV-1-infected ducklings reveals a complex interplay between viral replication, metabolic suppression, and immune activation. The peak in viral titer at 24 hpi coincides with a significant inhibition of oxidative phosphorylation and lipid metabolism, which may lead to liver enlargement. Integrating COG classification, GO analysis, and KEGG pathway enrichment with viral replication data highlights key pathways contributing to liver pathology. The activation of the Jak–STAT, RIG-I-like receptor, and Toll-like receptor signaling pathways underscores the dual role of immune responses in both controlling the virus and contributing to liver pathology. These findings enhance our understanding of DHAV-1 pathogenesis and may inform the development of therapeutic strategies to combat this viral infection.

## Data Availability

The original contributions presented in the study are deposited in the China National Center for Bioinformation (https://ngdc.cncb.ac.cn/gsa/), accession number CRA038079.
